# Less but better: cardioprotective lipid profile of patients with GCK-MODY despite lower HDL cholesterol level

**DOI:** 10.1007/s00592-014-0567-1

**Published:** 2014-02-19

**Authors:** Wojciech Fendler, Manfredi Rizzo, Maciej Borowiec, Beata Malachowska, Karolina Antosik, Agnieszka Szadkowska, Maciej Banach, Malgorzata Urbanska-Kosinska, Magdalena Szopa, Maciej Malecki, Wojciech Mlynarski

**Affiliations:** 1 Department of Pediatrics, Oncology, Hematology and Diabetology, Medical University of Lodz, 36/50 Sporna Str., 91-738 Lodz, Poland; 2 Department of Clinical Medicine and Emerging Diseases, University of Palermo, Palermo, Italy; 3 Department of Clinical Genetics, Medical University of Lodz, Lodz, Poland; 4 Department of Hypertension, WAM University Hospital in Lodz, Medical University of Lodz, Lodz, Poland; 5 Department of Pediatrics, Regional Specialist Hospital, Zielona Gora, Poland; 6 Department of Metabolic Diseases, Collegium Medicum Jagiellonian University of Cracow, Krakow, Poland

**Keywords:** MODY, Monogenic diabetes, Lipid subpopulations

## Abstract

Patients with diabetes caused by single-gene mutations generally exhibit an altered course of diabetes. Those with mutations of the glucokinase gene (*GCK*-MODY) show good metabolic control and low risk of cardiovascular complications despite paradoxically lowered high-density lipoprotein (HDL) cholesterol levels. In order to investigate the matter, we analyzed the composition of low-density lipoprotein (LDL) and HDL subpopulations in such individuals. The LipoPrint^©^ system (Quantimetrix, USA) based on non-denaturing, linear polyacrylamide gel electrophoresis was used to separate and measure LDL and HDL subclasses in fresh-frozen serum samples from patients with mutations of glucokinase or *HNF1A*, type 1 diabetes (T1DM) and healthy controls. Fresh serum samples from a total of 37 monogenic diabetes patients (21 from *GCK*-MODY and 16 from *HNF1A*-MODY), 22 T1DM patients and 15 healthy individuals were measured in this study. Concentrations of the small, highly atherogenic LDL subpopulation were similar among the compared groups. Large HDL percentage was significantly higher in *GCK*-MODY than in control (*p* = 0.0003), T1DM (*p* = 0.0006) and *HNF1A*-MODY groups (*p* = 0.0246). Patients with *GCK*-MODY were characterized by significantly lower intermediate HDL levels than controls (*p* = 0.0003) and T1DM (*p* = 0.0005). Small, potentially atherogenic HDL content differed significantly with the *GCK*-MODY group showing concentrations of that subfraction from control (*p* = 0.0096), T1DM (*p* = 0.0193) and *HNF1A*-MODY (*p* = 0.0057) groups. Within-group heterogeneity suggested the existence of potential gene–gene or gene–environment interactions. *GCK*-MODY is characterized by a strongly protective profile of HDL cholesterol subpopulations. A degree of heterogeneity within the groups suggests the existence of interactions with other genetic or clinical factors.

## Introduction

Although dyslipidemia is considered as a traditional risk component for the metabolic syndrome, its qualitative aspects, genetically determined subfractions and variation in proatherogenic tendency have generated renewed interest and debate [1]. Different cholesterol concentrations were reported in diabetes caused by single-gene mutations in children and young adults [2, 3]. These studies reported differences in apolipoprotein and HDL (high-density lipoprotein) cholesterol levels. However, patients with diabetes caused by mutations of the glucokinase gene (*GCK*-MODY), who generally do not experience increased risk of cardiovascular (CV) events despite being diagnosed with diabetes, were shown to have lower levels of HDL than healthy controls [3]. This somewhat counterintuitive observation leads us to investigate the detailed composition of low-density lipoprotein (LDL) and HDL subpopulations in sera of adolescents and young adults with diabetes of autoimmune and monogenic background. A potential explanation would be that there are quantitative differences in the composition of lipid subfractions as was previously shown in the case of *HNF1A*-MODY and T2DM [2]. Since it is recommended that the patients with *GCK*-MODY should be treated with lifestyle modification and diet only, it is important to give an additional evidence that these diabetic patients are not at risk for CV complications. Should patients with *GCK*-MODY exhibit an altered composition of lipid subfractions, one may speculate that some of them would be candidates for the use of lipid-lowering agents.

## Methods

### Recruitment

The control group composed of young adults was selected from among healthy, non-obese parents of children treated for non-serious upper respiratory tract infections. Recruitment of the control group and all laboratory analyses were performed in the period of August 2012 to February 2013. The *GCK*-MODY group was selected from the nationwide database of the Polish Registry for Monogenic Diabetes [4]. Fresh samples were obtained from patients from previously reported patients with *GCK*-MODY [5, 6] at diagnostic or follow-up visits scheduled during the study period and were stored in -80 °C until analysis. The T1DM group was planned to match the size of the *GCK*-MODY group and was recruited from among the previously reported patients, with their samples collected during follow-up visits between August 2012 and February 2013 [7]. Patients with *HNF1A*-MODY were recruited by the Department of Metabolic Disorders in Cracow from individuals recruited during earlier studies [8]. The sample size of the MODY groups was determined by the availability of samples that were frozen directly after serum separation and did not undergo any freeze–thaw cycles to avoid decay of lipid subpopulations. The study was performed in accordance with the ethical standards of the responsible committee on human experimentation (institutional and national) and with the Helsinki Declaration of 1975, as revised in 2000 and 2008. All patients gave their written consent for participation in the project, and its protocol has been approved by the Institutional Bioethics Committee of the Medical University of Lodz.

### Lipid assays

All study individuals were instructed to fast at least 8 h prior to lipid profile assessment. Serum samples used in the study were frozen immediately after centrifugation and shipped with dry ice to the laboratory in Palermo (Italy). Non-denaturing, linear polyacrylamide gel electrophoresis was used to separate and measure LDL subclasses, with the LipoPrint^©^ system (Quantimetrix Corporation, Redondo Beach, CA, USA) [9]. From each serum sample, 25 μl was taken to be mixed with 200 μl (for LDL, IDL and VLDL) or 300 μl (for HDL) of LipoPrint loading gel and loaded on the upper part of the 3 % polyacrylamide gel. After 30 min of photopolymerization at room temperature, electrophoresis was performed for 60 (LDL) or 50 (HDL) min at 3 mA. Each electrophoresis chamber included two quality controls. After scanning, electrophoretic mobility and the area under the curve were calculated qualitatively and quantitatively. The digital image is analyzed using software provided by the producer—its computational algorithm program calculates the cholesterol level for each of the lipoprotein fractions and subfractions on the basis of area under the curve measurement for each of the separated fractions, as performed in other studies using the LipoPrint assay [2, 10]. LDL subclasses were distributed as seven bands: LDL-1 and LDL-2 defined as large LDL and LDL-3 to LDL-7 defined as small LDL [11]. HDL subpopulations were distributed as ten bands: HDL-1, HDL-2 and HDL-3 defined as large HDL; HDL-4, HDL-5, HDL-6 and HDL-7 defined as intermediate HDL; HDL-8, HDL-9 and HDL-10 comprising the small HDL portion [12]. The cholesterol concentration of each of the VLDL, IDL, LDL and HDL subpopulations was determined by multiplying the relative area under the curve of each band by respective lipoprotein concentration.

Analysis of variance of log-transformed cholesterol subpopulation concentrations (ANOVA) was used in univariate comparisons. Due to the expected differences in cholesterol and triglyceride concentrations across the groups, we used % content of each of the subpopulations for standardized profile assessment across the compared groups. General linear regression models were used to compare lipid subpopulation levels after adjustment for age, sex and body mass index (BMI). Post hoc testing was performed using the Tukey’s HSD test [13]. *p* values lower than 0.05 were considered as statistically significant. Statistical analysis was conducted using Statistica 10.0 software (StatSoft, Tulsa, OK, USA).

## Results

Overall, we were able to obtain 42 fresh serum samples from patients with monogenic diabetes: 22 from *GCK*-MODY and 16 from *HNF1A*-MODY groups. A group of 22 eligible individuals with T1DM and 15 healthy individuals agreed to undergo the lipid profiling experiment. One serum sample from the *GCK*-MODY group had to be discarded due to hemolysis, leaving a final sample size of 79 patients. The studied group characteristics are provided in Table 1.

**Table 1:** Clinical characteristics of the studied groups and lipid profile data

Variable	*GCK*-MODY	*HNF1A*-MODY	T1DM	Controls	*p* value in univariate analysis	*p* value adjusted for age, sex and BMI
Sex M/F	12/9	2/14	13/9	2/13	**0**.**0014**	NA
Age at examination (years)	25.05 (20.48-30.70)	35.00 (22.0–44.0)	20.47 (11.00–27.79)	29.72 (23.50–38.78)	**0**.**0068**	NA
Type of treatment	2—insulin, 5—oral agents, 14—diet	3—diet, 3—oral agents, 8—insulin, 2—no data	22—insulin	Not treated	**<0**.**0001**	NA
Duration of diabetes (years)	1.30 (0.22–2.85)	7.50 (2.50–14.00)	12.70 (0.60–19.65)	NA	**0**.**0346**	NA
BMI (kg/m^2^)	24.00 (22.20–25.10)	21.12 (19.61–23.88)	23.15 (18.39–27.47)	23.39 (21.88–23.88)	0.3979	NA
Glycated hemoglobin level (HbA1c) (%)	6.25 (6.00–6.45)	7.1 (5.50–7.90)	7.30 (6.80–7.90)	NA	0.0990	NA
Total cholesterol level (mg/dl)	132.0 (117.0–176.0)	184.0 (158.5–229.5)	163.5 (143.0–172.0)	184.0 (17.5–197.0)	**0**.**0164**	0.3121
HDL cholesterol (mg/dl)	45.0 (36.0–54.0)	51.0 (42.0–55.5)	54.0 (45.0–64.0)	60.0 (43.0–79.0)	**0**.**0364**	0.0698
Triglycerides (mg/dl)	157.0 (93.0–176.0)	119.5 (81.5–165.5)	78.0 (48.0–98.0)	108.0 (65.0–166.0)	**0**.**0035**	0.0536
LDL cholesterol (mg/dl)	65.6 (46.8–88.2)	108.9 (79.0–152.4)	92.2 (73.0–104.8)	86.4 (70.2–101.4)	**0**.**0105**	0.0743
VLDL (%)	21.0 (17.4–23.7)	17.6 (15.4–19.4)	18.3 (15.0–23.1)	13.8 (12.2–16.7)	**0**.**00028**	**0**.**0196**
IDL-C (%)	11.1 (8.6–13.6)	9.0 (7.5–10.7)	6.8 (5.8–8.7)	5.7 (5.0–6.9)	<**0**.**0001**	<**0**.**0001**
IDL-B (%)	6.3 (5.4–7.6)	7.2 (6.0–8.3)	5.8 (4.7–7.2)	6.2 (5.0–6.6)	**0**.**0388**	**0**.**0339**
IDL-A (%)	6.1 (4.1–9.6)	8.6 (7.0–9.9)	6.4 (3.7–8.3)	8.0 (7.2–10.5)	**0**.**0320**	**0**.**0959**
Large LDL (%)	17.9 (14.6–24.8)	23.0 (15.1–25.0)	30.0 (25.3–31.9)	31.0 (28.3–34.2)	<**0**.**0001**	<**0**.**0001**
Small LDL (%)	1.4 (0.0–2.4)	1.3 (0.0–2.0)	2.0 (1.1–5.7)	1.0 (0.0–1.5)	0.2230	0.6280
Large HDL (%)	53.7 (45.0–67.3)	46.5 (42.7–48.9)	46.2 (39.4–50.8)	44.8 (28.0–51.5)	**0**.**0373**	<**0**.**0001**
Intermediate HDL (%)	38.7 (28.6–45.4)	40.6 (38.6–42.3)	44.1 (38.9–48.9)	47.0 (41.0–51.5)	**0**.**0068**	<**0**.**0001**
Small HDL (%)	6.6 (2.7–10.6)	12.6 (12.1–14.6)	10.3 (8.6–11.9)	10.4 (8.2-14.6)	**0**.**0036**	**0**.**0016**

Data are presented as medians and interquartile ranges or number of patients for sex and treatment proportion. Differences significant in adjusted, post hoc between-group comparisons are described within the text. Glycated hemoglobin was not measured within the healthy control group

Variables that differed significantly between the groups with a *p* level <0.05 had their *p* values presented in bold

*GCK* glucokinase, *HNF1A* hepatocyte nuclear factor-1 alpha, MODY maturity onset diabetes of the young, *ANOVA* analysis of variance, *NA* not applicable

**Fig. 1:**
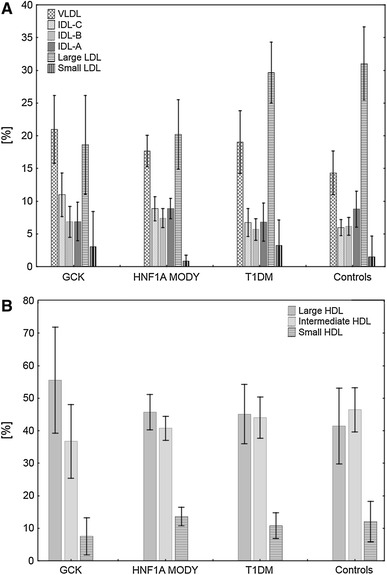
VLDL, IDL, LDL (**a**) and HDL (**b**) subpopulations in different types of diabetes. *GCK* glucokinase, *HNF1A* hepatocyte nuclear factor-1 alpha, *MODY* maturity onset diabetes of the young, *T1DM* type 1 diabetes

After adjustment for sex distribution, age at examination and BMI, significant differences between respective groups were noted in levels of very-low-density lipoprotein (VLDL), intermediate-density lipoproteins (IDL)-C and IDL-B, large LDL and all three HDL fractions (Table 1). Patients with *GCK*-MODY exhibited significantly higher VLDL levels than controls (*p* = 0.0004). VLDL levels in controls were also lower than in T1DM (*p* = 0.0283). Patients with *GCK*-MODY had higher IDL-C levels than control (*p* = 0.0002), T1DM (*p* = 0.0002) and *HNF1A*-MODY groups (*p* = 0.0044). IDL-B levels did not show significant differences in post hoc comparisons between any of the analyzed groups. Levels of the large LDL subpopulation were lower in both *GCK*- and *HNF1A*-MODY groups than in controls (*p* = 0.0002 and 0.0002, respectively) and T1DM (*p* = 0.0002 and *p* = 0.0010, respectively). Levels of the highly atherogenic small LDL subpopulation were similar among the compared groups (Fig. 1a). Large HDL percentage was significantly higher in *GCK*-MODY than in control (*p* = 0.0003), T1DM (*p* = 0.0006) and *HNF1A*-MODY groups (*p* = 0.0246). Patients with *GCK*-MODY were characterized by significantly lower intermediate HDL levels than controls (*p* = 0.0003) and T1DM (*p* = 0.0005). Small HDL content differed significantly between the groups, with the *GCK*-MODY group shown to have lower levels of this subfraction from the control (*p* = 0.0096), T1DM (*p* = 0.0193) and *HNF1A*-MODY groups (*p* = 0.0057) (Fig. 1b).

**Fig. 2:**
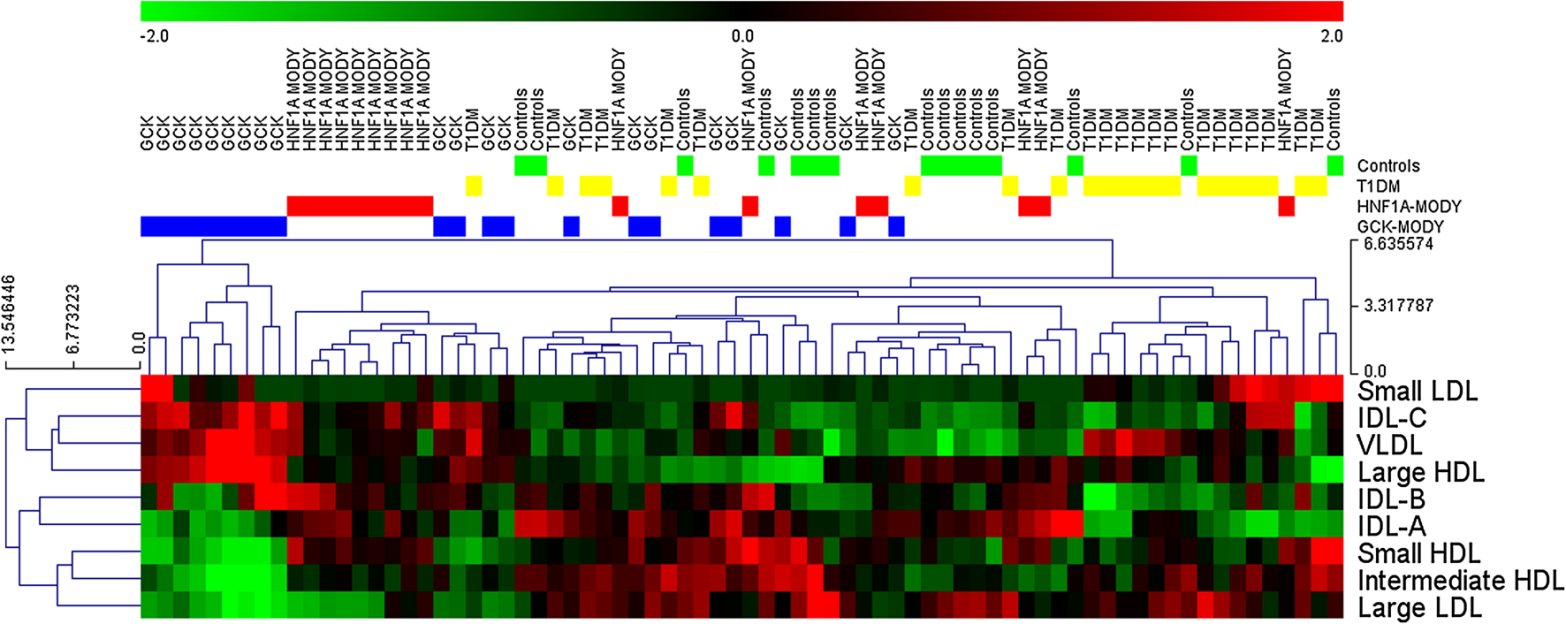
Hierarchical clustering plot of lipid profiles. Lipid subfraction levels were standardized across the samples, and Euclidean distance was used to visualize within- and between-group differences. Although the lipid profiles of the majority of patients from the *HNF1A*- and *GCK*-MODY groups showed within-group similarities, considerable heterogeneity is evident, which suggests an overlapping effect of environmental and/or other genetic factors

**Fig. 3:**
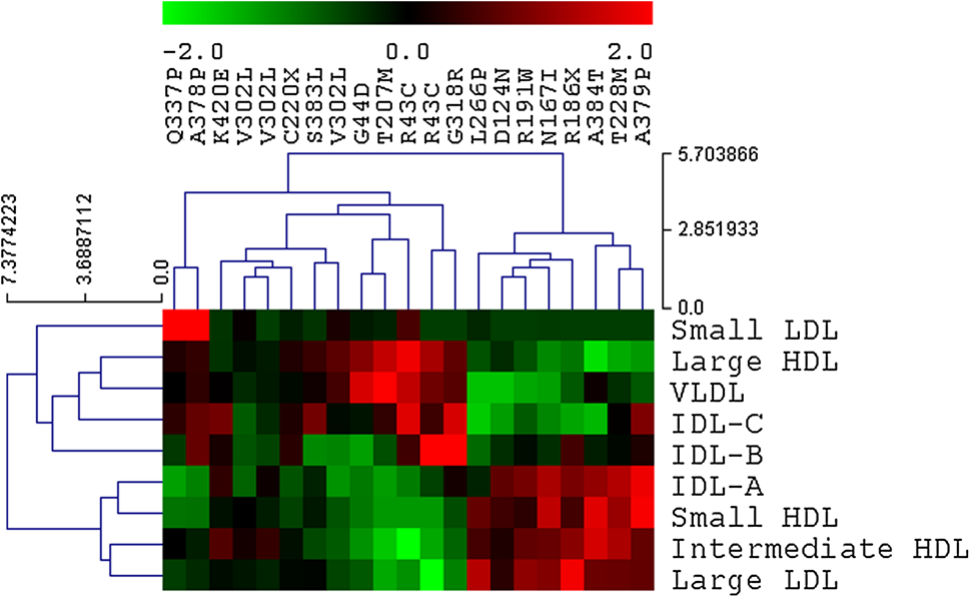
Hierarchical clustering of lipid profiles of patients diagnosed with *GCK*-MODY. Two mutations were present in more than one individual: R43C and V302L. In both cases, in carriers of such mutations similarities between lipid subfraction profiles were noted, suggesting a strong, mutation-specific effect

The patients age was not associated with significant changes in either the most atherogenic small LDL (*r* = −0.1339, *p* = 0.2803) or HDL (*r* = 0.0414, *p* = 0.7404) fractions. Profile analysis by hierarchical clustering showed evident heterogeneity despite statistically significant differences between the groups. Among patients with *GCK*- or *HNF1A*-MODY, nine individuals in both groups showed considerable similarities of their lipid profile, suggesting the existence of a MODY-specific effect (Fig. 2). In-depth analysis of patients with *GCK*-MODY showed that patients with identical mutations clustered more tightly together than individuals with other types of *GCK* gene alterations (Fig. 3). However, considerable variability was noted between patients with *GCK*-MODY, suggesting that the clinical phenotype of *GCK*-MODY may be strongly modulated also by other genetic or environmental factors.

The observed differences of lipid profiles were not associated with method of treatment, sex, age or diabetes duration. Glycated hemoglobin levels did not correlate significantly with lipid subpopulation percentages in the whole studied group (all −0.25 < *r*< 0.25 with *p* > 0.15). Among patients with type 1 diabetes, HbA1c levels showed non-significant correlations with small LDL and small HDL levels (*r* = 0.46, *p* = 0.08 and *r* = 0.48, *p* = 0.07, respectively). This lack of association may have been caused by good metabolic control in the whole studied group and small sample size of the subgroups with the poorest metabolic control.

## Discussion

Results of our study showed that individuals with *GCK*-MODY exhibit a strongly protective profile HDL cholesterol (high concentration of large HDL and low levels of intermediate and small HDL subpopulation) and provide a reference material for further studies on the subject. While constitutively moderately elevated glycemia observed in these patients probably is a major factor contributing to the low frequency of CV complications [14], the observed lipid profile may also be important. The observed lack of correlation of diabetes duration with profiles of the atherogenic LDL and HDL subpopulations, in combination with the non-progressive phenotype of *GCK*-MODY, supports the hypothesis of a disease-specific profile of serum lipids. Similarities noted among *HNF1A*-MODY also showed that the presence of a major genetic determinant of disease (a dominant mutation in case of MODY) may exert a strong effect on the lipid profile and contribute indirectly toward an altered risk of CV complications.

The degree of variability of lipid profiles among individuals with monogenic diabetes was considerable as evidenced by the incomplete clustering of particular groups (Figs. 2, 3). Given the presence of a strong genetic modifier responsible for the development of diabetes, this may seem somewhat surprising as the phenotypical profile was expected to be more uniform within the groups. However, in view of earlier reports about the interactions between common genetic variants and pathogenic mutations in *GCK*-MODY [15], a similar effect could well be the reason behind this heterogeneity. Such effects could be dependent on polymorphic variants of *GCKR* or *G6PC2* genes, both of which were shown to affect lipid and glucose metabolism [16]. However, given the abundance of clinical and genetic factors affecting cholesterol level, further large-scale studies supported by functional in vitro analyses are necessary to identify major contributors.

Although the study does not definitely resolve the issue of all possible alterations of lipid profile among patients with monogenic diabetes, it does contribute to the field of atherogenicity as the first report to show cardioprotective HDL subpopulation profiles in young adults with *GCK*-MODY. This group also exhibited lower concentrations of LDL and elevated VLDL in comparison with healthy controls and T1DM. As this may seem to be atherogenic, since large LDLs are generally considered as neutral or even protective [17], further studies into the LDL/HDL profile discrepancies are necessary to quantify the relative effects of either cholesterol type and their respective subpopulations. However, it was already reported that the Lp (a) lipoprotein, which is a known CV risk factor, is associated with LDL-2 levels, which were categorized as large LDLs in our analyses and were in fact shown to be present in significantly lower concentrations in patients with *GCK*-MODY group than in other studied groups [18].

We were, however, unable to avoid some limitations. Most importantly, the number of available patients was associated with a rigorous sample collection program introduced to avoid any methodological bias that would be introduced by repeated freezing and thawing of the sera [19]. In contrast to earlier studies investigating atherogenicity, we did not evaluate C-reactive protein (CRP) levels [20]. This was considered as unnecessary, as patients with *HNF1A*-MODY were previously reported to exhibit CRP levels near detection limit, while *GCK*-MODY or T1DM patients do not differ in that respect [21]. A similar rationale was behind our decision of not testing apolipoprotein M (ApoM) level. Patients with *HNF1A*-MODY were reported to have lower levels of ApoM, than those with T1DM [22] and similar to non-diabetic controls. As the diagnostic utility of the above markers, and according to a recent paper by Steele et al. [23] HbA1c as well, is sufficient in terms of discriminating between particular types of monogenic diabetes, we did not attempt to evaluate lipid subfractions as diagnostic tools or compare them with these markers but rather to ascertain the potential for atherogenic lipid profiles of the patients’ sera.

Impaired metabolic control could be another factor promoting dyslipidemia, but as the studied group was generally very well controlled in terms of HbA1c levels (median 6.5; 25–75 %, 5.95–7.55 %), it was impossible to precisely analyze or adjust for this factor without serial measurements or a group of individuals with worse metabolic control. Although some patients with monogenic diabetes caused by *HNF1A* or *GCK* mutations may show poor metabolic control, this is usually due to comorbidity with type 1 or type 2 diabetes [23, 24], making it impossible to study the impact of high HbA1c in “pure” monogenic diabetes.

It is a generally accepted fact that patients with *GCK*-MODY have lower triglyceride levels than controls and patients with other causes of diabetes [3, 25, 26]. It seemed possible that in some patients with *GCK*-MODY, high levels of triglycerides were related to hyperglycemia at the time of blood withdrawal, but none of the studied patients exhibited signs of ketoacidosis. Moreover, their HbA1c levels measured during the study, as well on the next follow-up (data not shown), did not hint at any evidence of a hyperglycemic episode. Unfortunately, we were unable to measure blood glucose levels to ascertain whether elevated blood glucose was the reason for unusually high triglyceride concentrations among patients with *GCK*-MODY. It is, however, now known that metabolic control in *GCK*-MODY is remarkably stable, regardless of treatment [27]. These considerations lead us to conclude that the possibility of a hyperglycemic cause of high triglycerides in our group was highly unlikely, suggesting a different cause of hypertriglyceridemia present in a small subset of patients with *GCK*-MODY.

Heterogeneity of lipid profiles among individuals with different types of monogenic diabetes was not associated with the type of treatment or any discernible clinical pattern. This suggests that an overlap of major genetic factors and common variants may have been in order, similarly as was the case for metabolic control in *GCK*-MODY individuals [15]. One could also speculate that the observed differences may be method-specific, as we performed the analysis using the LipoPrint electrophoresis-based assay. However, this technique was selected as it was shown to provide accurate results, was validated against NMR spectroscopy and other electrophoretic measurements [9, 28] and is less cumbersome in terms of application. Unfortunately, the heterogeneity of lipid subpopulation assessment methods makes it difficult to perform pooled analyses as discrimination between the different subpopulation levels may vary depending on the methodology used in particular studies, which has resulted in a number of seemingly conflicting reports on the role of specific subpopulations of HDL on the risk of CV events [29].

Other limitations arose from the distributed, multicenter nature of the monogenic diabetes registry study. We were unable to evaluate the patients uniformly in terms of cardiovascular risk scores by measuring coronary artery calcification (CAC) or evaluating other risk factors in a standardized manner. We are aware that calcification of coronary arteries was already shown to be frequent in patients with type 1 diabetes, although studies that focus on the subject generally cover patients with worse metabolic control and long-lasting diabetes [30, 31]. In our group, patients with monogenic diabetes had very short duration of diabetes and all groups showed low HbA1c levels, but we cannot exclude that some of them may in fact have had higher CAC scores. All studied patients were reportedly non-smokers, and none of them have experienced any cardiovascular events and were not using any lipid-lowering agents at the time of sample collection. Another source of bias could be a different duration of sample storage, as even in temperatures of −70 °C were reported as likely to exert an impact on HDL level [32]. Even though this did not corroborate with other studies on the matter [33], we were aware that storage duration and conditions might have been a factor affecting the analysis. To correct for this particular effect, data were analyzed as relative values against total concentrations of high- or low-density lipoproteins. It is possible that non-identical decay rates could thus affect the resultant profiles. However, given that the most significant differences were noted between the *GCK*-MODY and the T1DM and control groups, we did not consider this as a major cause of the observed differences, as short-term storage was shown not to affect the results of the Quantimetrix LipoPrint assay [9].

The final hypothesis generated by the results shown in this paper may be related to the emerging group of drugs termed as glucokinase activators. Since activation of glucokinase through the *GCKR* was reported to elevate triglyceride levels [34], one can expect that stimulation of glucokinase with pharmacological agents may alter the lipid profile in diabetic patients. Elevation of triglycerides was recently observed in an experimental study on mice treated with two glucokinase-activating compounds [35]. Although at this stage such effects in humans are difficult to foresee, the impact of drugs acting through glucokinase-dependent mechanisms warrants further investigation for adverse effects promoting the atherogenic process.

## Conclusions

*GCK*-MODY is characterized by strongly protective profile of HDL cholesterol subpopulations. A degree of heterogeneity within the groups suggests the existence of interactions with other genetic or clinical factors affecting the phenotype.
